# Outcomes of bisphosphonate and its supplements for bone loss in kidney transplant recipients: a systematic review and network meta-analysis

**DOI:** 10.1186/s12882-018-1076-1

**Published:** 2018-10-19

**Authors:** Yan Yang, Shi Qiu, Linghui Deng, Xi Tang, Xinrui Li, Qiang Wei, Ping Fu

**Affiliations:** 1 Kidney Research Laboratory, Division of Nephrology, National Clinical Research Center for Geriatrics, West China Hospital, Sichuan University, No. 37, Guoxue Alley, Chengdu, Sichuan People’s Republic of China 610041; 2 Department of Nephrology, The First People’s Hospital of Changzhou, The Third Affiliated Hospital of Soochow University, Changzhou, Jiangsu People’s Republic of China 213000; 3 Department of Urology, Institute of Urology, West China Hospital, Sichuan University, Chengdu, Sichuan People’s Republic of China; 4 Stroke Clinical Research Unit, Department of Neurology, West China Hospital, Sichuan University, Chengdu, Sichuan People’s Republic of China

**Keywords:** Kidney transplant, Bisphosphonates, Bone mineral density, Network meta-analysis

## Abstract

**Background:**

Mineral bone disease constitutes a common complication of post-kidney transplantation, leading to great disability. As there is no consensus on the optimal treatment for post-kidney transplant recipients (KTRs), we aimed to evaluate the efficacy and safety of bisphosphonate and its combined therapies.

**Methods:**

We incorporated relevant trials to perform a network meta-analysis from direct and indirect comparisons. We searched PubMed, Embase and the CENTRAL and the reference lists of relevant articles up to August 1, 2017, for randomized controlled trials. The primary outcome was bone mineral density (BMD) change at the femoral neck and the lumbar spine.

**Results:**

From a total of 864 citations, 18 randomized controlled trials with a total of 1200 participants were included. Five different regimens were considered. Bisphosphonate plus calcium revealed a significant gain in percent BMD change than calcium alone at the femoral neck (mean difference (MD), 5.83; 95% credible interval (CrI), 1.61 to 9.27). No significant difference was detected when restricting to absolute terms. At the lumbar spine, bisphosphonate and calcium with or without vitamin D analogs outperformed calcium solely (MD, 0.07; 95% CrI, 0.00 to 0.13; MD, 0.06; 95% CrI, 0.02 to 0.09). Compared to calcium with vitamin D analogs, adding bisphosphonate was associated with marked improvement (MD, 0.03; 95% CrI, 0.00 to 0.05). Considering percent terms, combination of bisphosphonate with calcium and vitamin D analogs showed greater beneficial effects than calcium alone or with either vitamin D analogs or calcitonin (MD, 10.51; 95% CrI, 5.92 to 15.34; MD, 5.48; 95% CrI, 2.57 to 8.42; MD, 6.39; 95% CrI, 0.55 to 12.89). Both bisphosphonate and vitamin D analogs combined with calcium displayed a notable improvement compared to calcium alone (MD, 7.24; 95% CrI, 3.73 to 10.69; MD, 5.02; 95% CrI, 1.20 to 8.84).

**Conclusions:**

Our study suggested that additional use of bisphosphonate was well-tolerated and more favorable in KTRs to improve BMD.

**Electronic supplementary material:**

The online version of this article (10.1186/s12882-018-1076-1) contains supplementary material, which is available to authorized users.

## Background

Since kidney transplantation (KT) became an effective treatment of patients with end-stage renal disease (ESRD), clinicians have paid more attention to complications of kidney transplant recipients (KTRs). Post-transplantation bone disease which can result in serious disabilities and fractures has been observed among a large proportion of KTRs [1]. According to Naylor and colleagues [2], the 5-year cumulative incidence of fracture ranged from 0.85 to 27% after KT. Hence, prevention and treatment of bone disorders are of great importance to improve high-quality long-term survival of KTRs.

The etiology of transplant bone disease is multifactorial and most KTRs have preexisting chronic kidney disease-mineral and bone disorders (CKD-MBD) [3]. Apart from these, glucocorticoid-induced suppression of bone formation, calcineurin inhibitors (CNIs) and persistent hyperparathyroidism are the most important risk factors for bone loss [4–6]. Postmenopausal status, prolonged immobilization, duration of CKD stage 5, smoking and presence of diabetes may also contribute to bone loss [4]. The Kidney Disease Improving Global Outcomes (KDIGO) guideline [7] suggested that “vitamin D, calcitriol/alfacalcidol, or bisphosphonates be considered for low BMD patients with stable graft function”, but it was derived from the very low quality of evidence.

Previous meta-analyses [8, 9] have demonstrated that bisphosphonates have favorable efficacy on bone mineral density (BMD), but questionable effect on the fracture risk. However, these studies did not examine the effect of co-intervention with calcium and/or vitamin D. Moreover, it is still uncertain that the optimal approach to prevent bone loss and whether it is need to use combined therapy. To obtain a better understanding on this issue, we performed a network meta-analysis (NMA). In this NMA, we systematically reviewed the literature and estimated relative treatment effects for all possible comparisons including bisphosphonates and co-intervention.

## Methods

### Search strategy

This systematic review is performed in keeping with Preferred Reporting Items for Systematic Reviews and Meta-analyses (PRISMA) guideline [10]. A comprehensive search was conducted in PubMed, Embase and the Cochrane Library Central Register of Controlled Trials (CENTRAL) by two independent investigators up to August 1st, 2017. The full search parameters for each database was outlined in Additional file 1. Referenced articles and systematic reviews were screened to maximize inclusion of pertinent data.

### Selection criteria

Only randomized controlled trials (RCTs) comparing bisphosphonate-treated and control groups of adult KTRs were included. The full-text original article with at least one interest outcome was finally involved. Two independent investigators (YY, QS) initially screened the citation titles and abstracts. Studies were excluded because of non-English text, combined transplantation. If duplicate studies from the identical authors were found, the reports were grouped together and only the publication with a complete data was used. Any discrepancies in the study inclusion were resolved by consulting the senior authors (TX).

### Data extraction and quality assessment

The independent reviewers (YY, SQ) used a standardized form to extract information from each eligible study. Data regarding study-, patient- and treatment-related characteristics and outcomes were extracted simultaneously. When relevant information was unclear or needed data was unavailable, attempts were made to obtain eligible data from the first or corresponding author of such studies. We assessed the validity of the NMA through a qualitative appraisal of study designs and methods. We executed the tool recommended by the Cochrane Collaboration to evaluate the risk of bias [11].

### Outcomes

The primary outcome was the BMD change (percent change and absolute change [in g/cm^2^]) at the lumbar spine and the femoral neck after successful KT. The secondary outcomes were overall fractures, all-cause mortality, graft loss, acute renal rejection, adverse events. The fractures occurred during reported follow-up time that identified by radiographs were used to calculate fracture incidence. Graft loss was regarded as a doubling of the baseline serum creatinine level or progressing to ESRD again. We used data from the longest complete follow-up, when the outcomes of different follow-up intervals were reported. If investigators published more than one report addressing the same population, we included the most comprehensive report.

### Data synthesis and statistical analysis

The pair-wise meta-analysis by the random-effects model was performed initially [12]. Results were expressed as mean difference (MD) with 95% confidence intervals (CI) for continuous outcomes (percent change and absolute change in BMD), while the odds ratio (OR) was used for dichotomous variables (fracture, all-cause mortality, graft loss, acute renal rejection, adverse events). The level of statistical significance was set at *P* < 0·05 and all statistical tests were two-sided. The statistical heterogeneity among studies was evaluated by the Cochran’s Q test and the I^2^ statistic. A *P* value of 0.05 or less for the Q test or an I^2^ greater than 50% was suggestive of substantial study heterogeneity.

We performed random-effects Bayesian network meta-analyses for indirect and mixed comparisons using Markov chain Monte Carlo methods in WinBUGS version 1.4.3 (MRC Biostatistics Unit) [13]. A Bayesian fixed-effect framework was deemed appropriate because of the limited number of studies supporting each edge in the network [13, 14]. We report the resultant effect as OR or MD with corresponding 95% credibility intervals (CrIs), which are the Bayesian analogue of 95% CIs. We estimated the relative ranking probability of each strategy and obtained the hierarchy of competing interventions using rankograms and surface under the cumulative ranking curve (SUCRA) [15]. The SUCRA index ranges between 0 (or 0%) and 1 (or 100%), where the treatments with highest and lowest SUCRA are considered to be the best and worst treatments, respectively.

To assess the presence of the inconsistency, we employed the node-splitting method, excluding one direct comparison at a time and estimating the indirect treatment effect for the excluded comparison. To check the assumption of consistency in the entire network, the design-by-treatment model was conducted [14]. If the total residual deviance and the effective number of parameters (pD) are almost the same, the network consistency is considered to be satisfied. We then performed sensitivity analysis and meta-regressions to explore important network inconsistency.

### Quality of evidence

The quality of evidence was rated according to the Grading of Recommendations, Assessment, Development and Evaluation (GRADE) methodology [16]. In this approach, direct evidence from RCTs starts at high quality and can be downgraded based on the risk of bias, indirectness, imprecision, inconsistency (or heterogeneity) and publication bias to levels of moderate, low and relatively low quality [17].

## Results

### Study characteristics

**Fig. 1:**
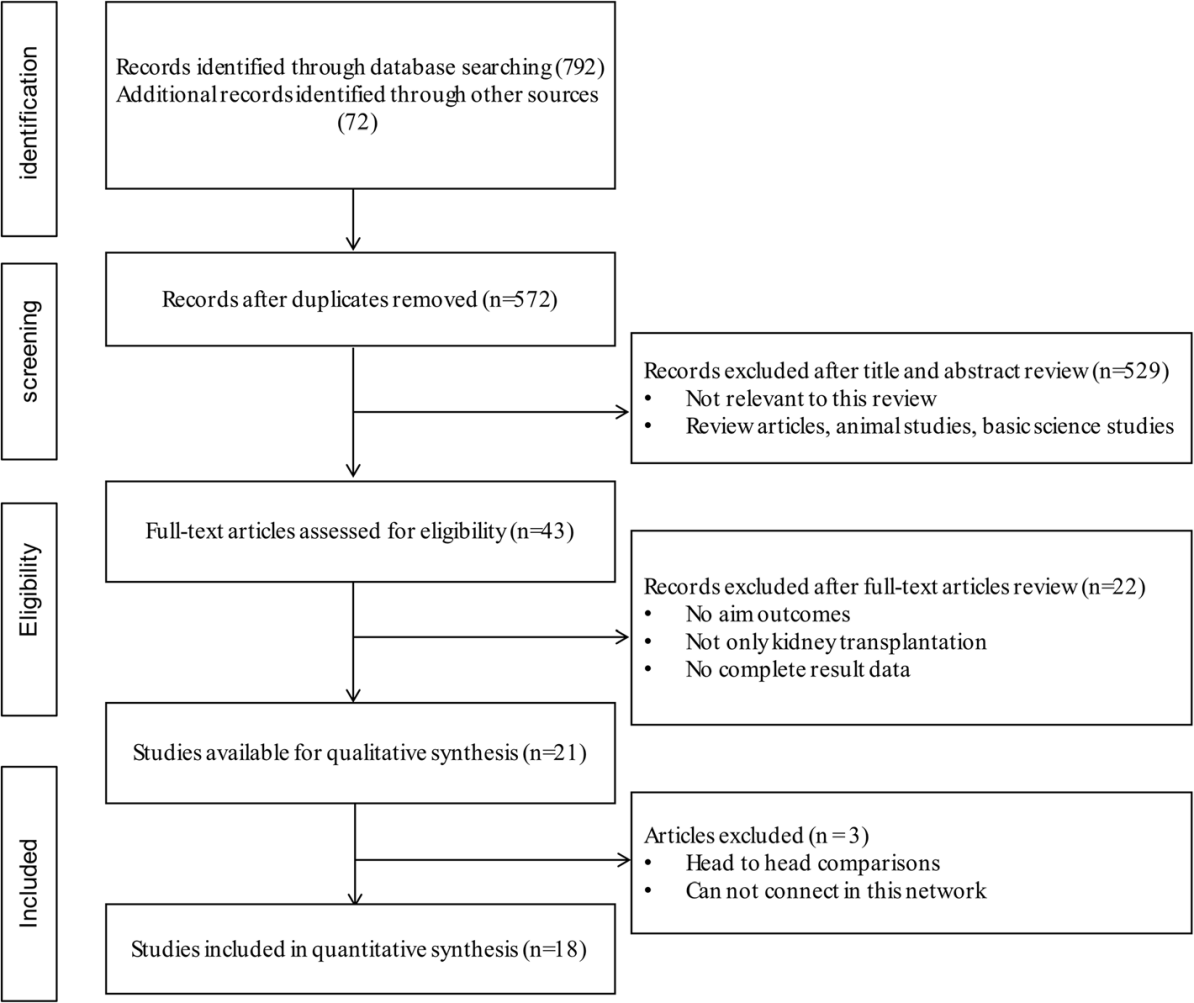
Flow chart of study identification and selection procedure

The PRISMA [10] flowchart depicting the electronic searching process is presented in Fig. 1. There are 864 potentially relevant articles identified through electronic and reference searches. According to title and abstract, 821 publications were excluded after the initial screening. A further 26 studies were excluded because they were not RCT, without available data of interest outcomes and lack of full-text. Overall, 18 RCTs (19 publications) [18–36] involving 1200 participants were included in this NMA. The studies were published between October 1998 and March 2015. The details of the interventions, baseline characteristics of the populations, follow-up period were outlined for NMA in Table 1. Most of the RCTs included both sexes, except one study [26] only included male patients and two studies [25, 27] did not mention. The number of patients allocated to each treatment ranged from 8 to 66, whereas patient follow-up duration ranged from 6 months to 3 years after first administration.

**Table 1:** Study characteristics

Study	Follow-up	Country	No. of Patients	Female/Male	Intervention	Bisphosphonate Administration	N	Co-intervention	Immunosuppression
Smerud 2012 [18]	12 months	Norway	129	30/99	ibandronate	3 mg i.v. (every 3 months)	66	PO calcium 500 mg tweice daily+ calcitriol 0.25 mcg daily	corticosteroids, MMF, CsA or FK506
					placebo		63		
Coco 2012 [19]	12 months	USA	42	15/27	risedronate	35 mg p.o. (weekly)	20	PO calcitriol 0.25 μg daily (with or without calcium)	corticosteroids, MMF, FK506, rapamycin
					placebo		22		
Torregrosa 2010 [20]	12 months	Spain	101	34/67	risedronate	35 mg p.o. (weekly)	52	PO calcium 1.5 g daily + vitamin D 400 IU daily	corticosteroids, FK506 with or without MMF
					no treatment		49		
Torregrosa 2011 [21]	12 months	Spain	39	13/26	pamidronate	30 mg i.v. (between day 7 and 10 after KT and 3 months post-KT)	24	PO calcium 1 g daily + cholecalciferol 800 IU daily	corticosteroids, MMF, CsA
					placebo		15		
Walsh 2009 [22]	24 months	UK	125	24/69	pamidronate	1 mg/kg i.v. (perioperatively and at month 1, 4, 8, 12)	65	PO calcium 500 mg daily + vitamin D 400 IU daily	corticosteroids, CsA
					no treatment		60		
Lan 2008 [23]	6 months	China	46	27/19	alendronate	70 mg p.o. (weekly)	23	PO calcium 800 mg daily + calcitriol 0.25 μg daily	corticosteroids, MMF, CsA
					no treatment		23		
Trabulus 2008 [24]	12 months	Turkey	64	19/40	alendronate	10 mg p.o. (daily)	13	PO calcium 1 g daily	corticosteroids, azathioprin or MMF, CsA or FK506
					alfacalcidol	0.5 μg p.o. (daily)	25		
					alendronate + alfacalcidol		17		
					no treatment		9		
Nayak 2007 [25]	6 months	India	50	NA	alendronate	35 mg p.o. (weekly)	27	PO calcium 1 g daily + vitamin D	NA
					no treatment		23		
El-Agroudy 2005 [26]	12 months	Egypt	60	0/60	alendronate	5 mg p.o. (daily)	15	PO calcium 500 mg daily	corticosteroids, CsA
					alfacalcidol	0.5 μg p.o. (daily)	15		
					calcitonin	100 μl intranasally (p.o.d and stopped for 1 month every 3 month)	15		
					no treatment		15		
Schwarz 2004 [27]	36 months	Austria	20	8/12	zoledronic acid	4 mg i.v. (week 2, month 3)	9	PO calcium 1 g daily	corticosteroids, MMF, CsA
					placebo		10		
Jeffery 2003 [28]	12 months	Canada	117	26/71	alendronate	10 mg p.o. (daily)	57	PO calcium 500 mg daily	corticosteroids, CsA, azathioprin or MMF
					calcitriol	0.25 μg p.o. (daily)	60		
Coco 2003 [29]	12 months	USA	72	28/31	pamidronate	60 mg i.v. (< 48 h after KT, 30 mg i.v. at months 1, 2, 3, 6)	36	calcium + calcitriol	corticosteroids, CsA or FK506
					no treatment		36		
Hass 2003 [30]	6 months	Austria	20	8/12	zoledronic acid	4 mg i.v. (week 2, month 3)	10	PO calcium 1 g daily	corticosteroids, MMF, CsA
					placebo		10		
Grotz 2001 [31]	12 months	Germany	80	24/48	ibandronate	1 mg i.v. (just before KT, 2 mg i.v. at month 3, 6, 9)	36	PO calcium 500 mg daily	corticosteroids, MMF, CsA
					no treatment		36		
Nam 2000 [32]	6 months	South Kore	50	21/29	pamidronate	30 mg i.v. (every 4 weeks)	15	PO calcium 500 mg daily	NA
					calcitriol	0.5 μg p.o. (daily)	15		
					no treatment		20		
Grotz 1998 [33]	12 months	Germany	46	17/29	clodronate	800 mg p.o. (daily) for 14 days, each followed by 75 days without treatment	15	PO calcium 500 mg daily	corticosteroids, CsA
					calcitonin	100 IU intranasally twice a day	16		
					no treatment		15		
Giannini 2001 [34]	12 months	Italy	40	13/27	alendronate	10 mg p.o. (daily)	20	PO calcium 500 mg daily + calcitriol 0.5 μg daily	corticosteroids, CsA with or without azathioprin
					no treatment		20		
Koc 2002 [35]	12 months	Turkey	35	10/25	alendronate	10 mg p.o. (daily)	8	PO calcium 1 g daily	corticosteroids, azathioprin, CsA
					calcitriol	0.5 μg p.o. (daily)	8		
					no treatment		8		
Torregrosa 2007 [36]	12 months	Spain	84	42/42	risedronate	35 mg p.o. (weekly)	39	PO calcium 2.5 g daily + vitamin D	corticosteroids, CsA or FK506, with or without MMF
					no treatment		45		

*KT*: kidney transplantation; *i.v*.: intravenous; p.o., *PO*: peros; *p.o.d*: per other day; *N*: numbers; *NA*: not available; *MMF*: mycophenolate mofetil; *CsA*: cyclosporine; FK506: tacrolimus; *AZA*: azathioprine; *mTOR*: mammalian target of rapamycin

As expected, most studies compared bisphosphonate with vitamin D analogs (cholecalciferol, alfacalcidol, calcitriol) or placebo. All patients in the trials included received co-intervention including calcium [24, 26–28, 30–33, 35], vitamin D analogs [19], or both. Bisphosphonate interventions encompassed pamidronate [21, 22, 29, 32], zoledronic acid [27, 30] and ibandronate [18, 31] that were administered intravenously, while clodronate [33], alendronate [23–26, 28, 34, 35] and risedronate [19, 20, 36] were given orally.

### Risk of Bias assessment result

The results from the risk of bias assessment are provided in Additional file 2. Details regarding trial methodology were unsatisfactory or incomplete for the majority of studies. Overall, there were 6 (32%) studies regarded as high risk of bias. Only 10 (53%) studies performed randomized sequence generation adequately. Furthermore, the risk of bias for concealment of treatment allocation was unclear in 10 (53%) studies. Only 4 (33%) studies explicitly reported blinding of participants and investigators, whereas the remaining studies were at high or unclear risk in this regard. The investigators attempted to blind outcome assessors in 6 (32%) studies, 3 studies did not make an effort to blind assessors, and the residual studies were unclear. When the results were summarized from at least 10 studies, the publication bias accessed via funnel plot. Comparison adjusted funnel plot showed no evidence of asymmetry (Additional file 2).

### Pairwise meta-analysis

Primary results of pairwise meta-analysis (direct comparisons) are summarized in Table 2. In terms of absolute change for the longest follow-up, adding bisphosphonate was associated with a marginal improvement compared to the combination of calcium and vitamin D analogs (femoral neck: MD, 0.36; 95% CI, 0.08 to 0.64; lumbar spine: MD, 0.38; 95% CI, 0.19 to 0.57). Bisphosphonate combined with calcium was significantly better than calcium alone (femoral neck: MD, 1.30; 95% CI, 0.92 to 1.68; lumbar spine: MD, 0.51; 95% CI, 0.20 to 0.82). Treatments with calcium alone displayed significantly lower absolute change at the femoral neck than combining with vitamin D analogs or calcitonin (MD, − 0.74; 95% CI, − 1.34 to − 0.14; MD, − 0.55; 95% CI, − 1.07 to − 0.03). When measured in percent terms, additional use of vitamin D analogs or bisphosphonate was significantly better than solely calcium (femoral neck: MD 1.53; 95% CI, 0.88 to 2.18; MD 1.14; 95% CI, 0.78 to 1.51; lumbar spine: MD 2.73; 95% CI, 1.95 to 3.51; MD 1.17; 95% CI, 0.80 to 1.54). Compared to calcium and vitamin D analogs, the combination of bisphosphonate and calcium showed significant improvement (femoral neck: MD, 1.55; 95% CI, 0.76 to 2.35; lumbar spine: MD, 1.53; 95% CI, 0.79 to 2.27). Bisphosphonate with calcium and vitamin D analogs also showed a significant gain at the lumbar spine compared to calcium and vitamin D analogs (MD, 1.32; 95% CI, 1.02 to 1.62).

**Table 2:** Summary effect size of pairwise and network meta-analysis

Comparisons	No. of directed trials (participants)	Pairwise meta-analysis mean differences (95% CI)	Network meta-analysis mean differences (95% CrI)	Heterogeneity I^2^	*P*-Value	Quality of evidence
Absolute BMD change at the femoral neck (536)						
Bis+Ca vs. Bis+Ca + Vit D	1 (29)	–	− 0.01 (− 0.32, 0.29)	–	–	Low
Bis+Ca vs. Ca	5 (167)	**1.3 (0.92, 1.68)**	0.19 (− 0.01, 0.38)	94.70%	0.000	Low
Bis+Ca vs. Ca + Vit D	2 (176)	0.26 (−0.04, 0.56)	0.06 (− 0.15, 0.26)	38.10%	0.184	Moderate
Bis+Ca vs. Ca + Calcitonin	2 (61)	0.21 (−0.29, 0.72)	0.06 (− 0.22, 0.36)	24.60%	0.249	Moderate
Bis+Ca + Vit D vs. Ca	–	–	0.20 (−0.14, 0.53)	–	–	Very low
Bis+Ca + Vit D vs. Ca + Vit D	4 (206)	**0.36 (0.08, 0.64)**	0.07 (−0.18, 0.30)	67.60%	0.026	Low
Bis+Ca + Vit D vs. Ca + Calcitonin	–	–	0.07 (−0.34, 0.46)	–	–	Very low
Ca vs. Ca + Vit D	2 (46)	**−0.74 (−1.34, − 0.14)**	−0.13 (− 0.38, 0.13)	0.00%	0.403	Low
Ca vs. Ca + Calcitonin	2 (60)	**−0.55 (−1.07, − 0.03)**	−0.12 (− 0.41, 0.19)	60.20%	0.113	Low
Ca + Vit D vs. Ca + Calcitonin	1 (30)	–	0.00 (−0.30, 0.34)	–	–	Low
Percent BMD change at the femoral neck (284)						
Bis+Ca vs. Bis+Ca + Vit D	1 (29)	–	−4.60 (−18.07, 7.67)	–	–	Low
Bis+Ca vs. Ca	4 (152)	**1.14 (0.78, 1.51)**	**5.83 (1.61, 9.27)**	91.10%	0.000	Moderate
Bis+Ca vs. Ca + Vit D	4 (46)	**1.55 (0.76, 2.35)**	−0.24 (5.62, 9.79)	96.10%	0.000	Low
Bis+Ca vs. Ca + Calcitonin	1 (31)	–	−0.04 (−19.65, 18.12)	–	–	Low
Bis+Ca + Vit D vs. Ca	–	–	10.43 (−2.64, 23.31)	–	–	Very low
Bis+Ca + Vit D vs. Ca + Vit D	–	–	4.35 (−2.29, 11.37)	–	–	Very low
Bis+Ca + Vit D vs. Ca + Calcitonin	–	–	4.56 (−18.36, 19.16)	–	–	Very low
Ca vs. Ca + Vit D	3 (51)	**−1.53 (−2.18, −0.88)**	−6.07 (− 17.09, 4.47)	79.30%	0.028	Low
Ca vs. Ca + Calcitonin	1 (30)	–	−5.87 (−20.01, 18.60)	–	–	Low
Ca + Vit D vs. Ca + Calcitonin	1 (30)	–	0.20 (−19.15, 19.61)	–	–	Low
Absolute BMD change at the lumbar spine (814)						
Bis+Ca vs. Bis+Ca + Vit D	1 (29)	–	−0.01 (−0.06, 0.04)	–	–	Low
Bis+Ca vs. Ca	5 (167)	**0.51 (0.20, 0.82)**	**0.06 (0.02, 0.09)**	0.00%	0.571	Moderate
Bis+Ca vs. Ca + Vit D	4 (176)	0.19 (−0.11, 0.49)	0.01 (−0.03, 0.06)	0.00%	0.866	Moderate
Bis+Ca vs. Ca + Calcitonin	2 (61)	0.49 (−0.02, 1.00)	0.05 (−0.01, 0.11)	24.60%	0.250	Moderate
Bis+Ca + Vit D vs. Ca	1 (30)	–	**0.07 (0.00, 0.13)**	–	–	Low
Bis+Ca + Vit D vs. Ca + Vit D	8 (484)	**0.38 (0.19, 0.57)**	**0.03 (0.00, 0.05)**	92.10%	0.000	Moderate
Bis+Ca + Vit D vs. Ca + Calcitonin	–	–	0.06 (−0.01, 0.15)	–	–	Very low
Ca vs. Ca + Vit D	2 (46)	−0.40 (− 0.99, 0.18)	−0.04 (− 0.10, 0.02)	0.00%	0.960	Moderate
Ca vs. Ca + Calcitonin	2 (60)	−0.04 (− 0.55, 0.47)	−0.01 (− 0.07, 0.06)	0.00%	0.874	Moderate
Ca + Vit D vs. Ca + Calcitonin	–	–	0.04 (−0.04, 0.12)	–	–	Very low
Percent BMD change at the lumbar spine (466)						
Bis+Ca vs. Bis+Ca + Vit D	–	–	−3.27 (−7.87, 0.84)	–	–	Very low
Bis+Ca vs. Ca	4 (152)	**1.17 (0.80, 1.54)**	**7.24 (3.73, 10.69)**	91.70%	0.000	Moderate
Bis+Ca vs. Ca + Vit D	2 (46)	**1.53 (0.79, 2.27)**	2.22 (−1.44, 5.73)	94.10%	0.000	Low
Bis+Ca vs. Ca + Calcitonin	1 (31)	–	3.13 (−2.51, 8.51)	–	–	Low
Bis+Ca + Vit D vs. Ca	–	–	**10.50 (5.92, 15.34)**	–	–	Very low
Bis+Ca + Vit D vs. Ca + Vit D	3 (145)	**1.32 (1.02, 1.62)**	**5.48 (2.57, 8.42)**	98.30%	0.000	Moderate
Bis+Ca + Vit D vs. Ca + Calcitonin	–	–	**6.39 (0.55, 12.89)**	–	–	Low
Ca vs. Ca + Vit D	2 (51)	**−2.73 (−3.51, −1.95)**	**−5.02 (−8.84, − 1.20)**	0.00%	0.373	Moderate
Ca vs. Ca + Calcitonin	1 (30)	–	−4.11 (−9.01, 0.72)	–	–	Low
Ca + Vit D vs. Ca + Calcitonin	–	–	0.91 (−4.38, 6.44)	–	–	Very low

Bis = bisphosphonate, Ca = calcium, Vit D = Vitamin D analogs, 95% CI = 95% Confidence Intervals, 95% CrI = 95% Credible Intervals. The mean difference with 95% CI or 95% CrI was used for continuous outcomes. Significant results are in bold. The Grading of Recommendations Assessment, Development and Evaluation (GRADE) approach specific to NMA served to assess the certainty in the evidence (quality of evidence) associated with specific comparisons, including direct, indirect, and final network meta-analysis estimates. The confidence assessment addressed the risk of bias (in individual studies), imprecision, inconsistency (heterogeneity in estimates of effect across studies), indirectness, and publication bias

### Network meta-analysis— Primary outcome

#### Change of BMD at the femoral neck

**Fig. 2:**
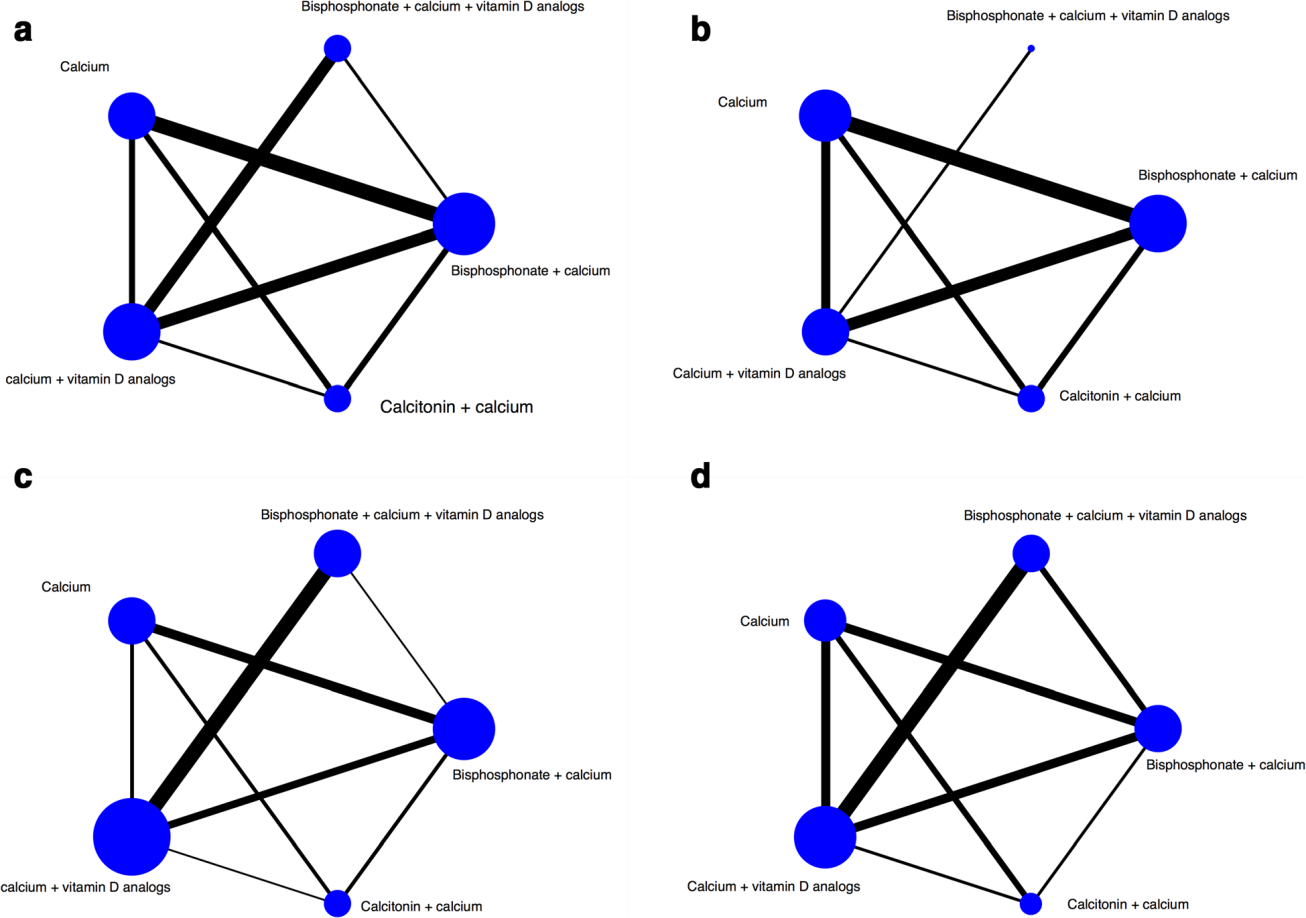
Network of eligible comparisons for primary outcome. The width of the lines is proportional to the number of trials comparing every pair of treatments, and the size of every circle is proportional to the number of randomly assigned participants (sample size). Network of included studies for all other outcomes is shown in Additional file 3. **a** Network of absolute change of BMD at the femoral neck; **b** Network of percent change of BMD at the femoral neck; **c** Network of absolute change of BMD at the lumbar spine; (**d**) Network of percent change of BMD at the lumbar spine.

**Fig. 3:**
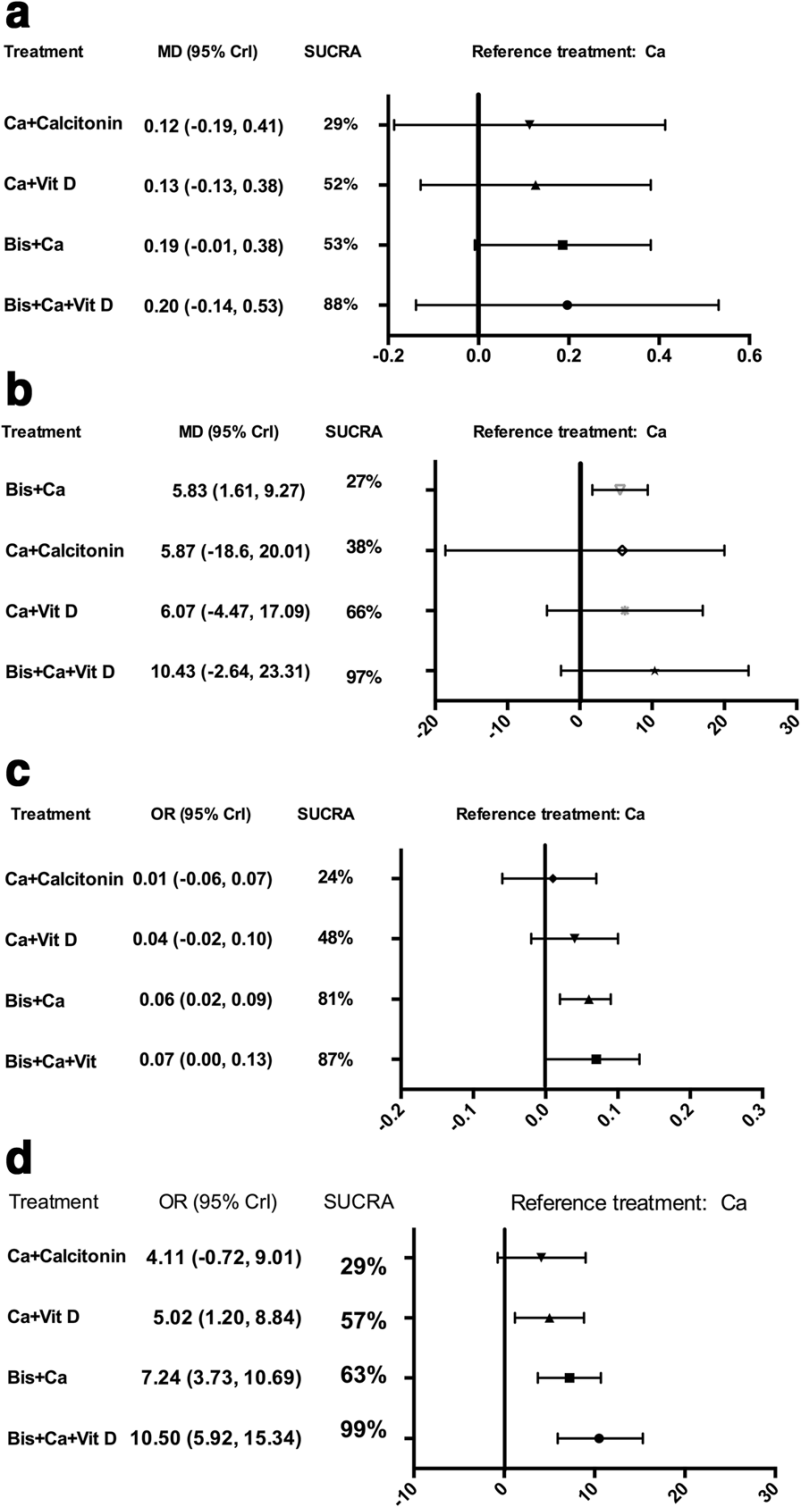
Forest plot of network meta-analysis results. Treatments are reported in order of efficacy ranking according to SUCRAs. All treatments are compared to calcium. **a** Summary mean difference and 95% credible intervals from network meta-analysis of absolute BMD change at the femoral neck; **b** Summary mean difference and 95% credible intervals from network meta-analysis of percent BMD change at the femoral neck; **c** Summary mean difference and 95% credible intervals from network meta-analysis of absolute BMD change at the lumbar spine; **d** Summary mean difference and 95% credible intervals from network meta-analysis of percent BMD change at the lumbar spine; MD: mean difference; CrI: credible intervals; SUCRA: surface under the cumulative ranking curve; Ca: calcium; Bis: bisphosphonate; Vit D: Vitamin D analogs.

Ten RCTs involving 536 adults evaluated the absolute change in BMD at the femoral neck. Figure 2 summarizes the network of direct evidence available for this outcome. No statistically significant difference was detected between each treatment groups. The SUCRA value for the regimens were 88%, 53%, 52%, 29%, 28% for bisphosphonate with calcium, bisphosphonate with calcium and vitamin D analogs, calcium with vitamin D analogs, calcitonin with calcium and calcium (Fig. 3a).

The result of percent terms was reported by 5 RCTs including 284 patients. Only bisphosphonate plus calcium revealed a significant gain in percent BMD change than calcium alone (MD, 5.83; 95% CrI, 1.61 to 9.27). No statistical difference was observed between other groups. Bisphosphonate combined with calcium and vitamin D analogs had the highest SUCRA value (97% Fig. 3b), followed by calcitonin with calcium (66%), bisphosphonate plus calcium (38%), calcium with vitamin D analogs (27%), and calcium only (22%).

### Change of BMD at the lumbar spine

14 RCTs including 814 participants provided data for comparison of absolute change in BMD at the lumbar spine. Bisphosphonate and calcium with or without vitamin D analogs outperformed calcium solely (MD, 0.07; 95% CrI, 0.00 to 0.13; MD, 0.06; 95% CrI, 0.02 to 0.09). We also observed that compared to calcium with vitamin D analogs, adding bisphosphonate was associated with marked improvement (MD, 0.03; 95% CrI, 0.00 to 0.05). The SUCRA value for each treatment formulations were as follows (Fig. 3c): bisphosphonate with calcium and vitamin D analogs (87%), bisphosphonate with calcium (81%), calcium plus vitamin D analogs (48%), calcitonin with calcium (24%) and calcium solely (10%).

Considering percent terms, the result analyzed using data from 7 trials (466 patients). Combination of bisphosphonate with calcium and vitamin D analogs showed greater beneficial effects than calcium alone or with either vitamin D analogs or calcitonin (MD, 10.51; 95% CrI, 5.92 to 15.34; MD, 5.48; 95% CrI, 2.57 to 8.42; MD, 6.39; 95% CrI, 0.55 to 12.89). Both bisphosphonate and vitamin D analogs combined with calcium displayed a notable improvement compared to calcium alone (MD, 7.24; 95% CrI, 3.73 to 10.69; MD, 5.02; 95% CrI, 1.20 to 8.84). As expected, bisphosphonate combined with calcium and vitamin D analogs had the highest SUCRA value (Fig. 3d 99%), followed by bisphosphonate with calcium (63%), calcium with vitamin D analogs (57%), calcitonin with calcium (29%), and calcium only (2%).

### Secondary outcomes

We did not observe a significant difference in the incidence of fractures from the direct comparisons and it could not connect to draw network geometries. All treatments have uncertain effects on all-cause mortality and graft loss metrics. Similarly, there were no statistical differences in the number of biopsy-proven acute rejections as well as adverse events among treatment groups. However, we found more adverse events happened in bisphosphonate and calcium than in calcium alone (OR, 5.41; 95% CrI, 1.15 to 25.49) from pairwise meta-analysis. Further details of the secondary outcome analyses are presented in Additional files 3 and 4.

### Network consistency

No evidence of small study effects based on funnel plot asymmetry was observed, but the number of studies included in each comparison was small. There was no evidence of inconsistency in the NMA when we applied the node-splitting approach. The total residual deviance for the outcomes of percent change (23.73, pD = 22) and absolute change (43.86, pD = 45) of BMD at the lumbar spine implied a good model fit, as well as percent change (25.22, pD = 26) and absolute change (24.36, pD = 24) at the femoral neck.

### Sensitivity analysis

For the sensitivity analyses, we used the full network for the primary outcome. In the first analysis, we investigated the different assumptions regarding the potential relationship between time and treatment effect, Bayesian NMA were repeated using the absolute change of BMD at the twelve-month follow-up period. We observed comparable results at the lumbar spine, adding bisphosphonate showed significant improvement than calcium alone or calcium with vitamin D analogs (Additional file 5: MD, 0.06; 95% CrI, 0.01 to 0.10; MD, 0.03; 95% CrI, 0.00 to 0.07). We also observed that bisphosphonate with calcium and vitamin D analogs outperformed calcium solely (MD, 0.07; 95% CrI, 0.01 to 0.15). At the femoral neck, bisphosphonate with calcium showed a significant preference than calcium alone (Additional file 5; MD, 0.23; 95% CrI, 0.02 to 0.46). The parameter estimates were consistent with the main analysis. We carried out separate meta-regressions to test the effect of length of trial, publication date and modes of administration. No evidence exists for an interaction between any of the trial characteristics assessed and the treatment effect.

### Quality of evidence

In general, there was no serious risk of bias, indirectness, inconsistency, or publication bias for any of the direct comparisons. In several comparisons, there was serious imprecision in summary estimate because the 95% credible interval crossed unity. The GRADE quality of evidence supporting the use of each treatment for the primary outcome was outlined in Table 2. According to GRADE, we had moderate confidence in estimates supporting the combination use of bisphosphonate or vitamin D analogs with calcium for improving BMD at the lumbar spine. We detected using bisphosphonate combined with vitamin D analogs and calcium considering BMD change at the lumbar spine with low quality evidence. There was very confidence in estimates supporting using calcitonin with calcium both at the lumbar spine and the femoral neck. Conceptually, there was no significant intransitivity.

## Discussion

This NMA was aimed to investigate the comparable efficacy and safety of bisphosphonate and its co-interventions for the post-transplantation bone disease. We found the combination of bisphosphonate, calcium and vitamin D analogs was the most effective to prevent bone and restore or improve BMD. However, the effects on fracture risk, adverse events, death, acute renal rejection and graft loss were still uncertain because of insufficient data and short follow-up time.

The current study revealed that only calcium prescription could not benefit KTRs from bone loss. We may suggest KTRs take calcium and vitamin D analogs orally because this NMA showed combination therapy of calcium and vitamin D analogs could improve BMD than calcium alone with moderate quality evidence. The result was supported by previous studies [37, 38]. Our work displayed that calcitonin with calcium seemed only better than calcium alone with low strength of evidence. Two RCTs [26, 33] involving 31 patients allocated to receive calcitonin, and incidence of hypocalcemia was reported so that we did not suggest giving calcitonin for KTRs. In our study, additional use of bisphosphonate could improve BMD changes at the lumbar spine and femoral neck. It was in accordance with previous analyses [9, 39], though they did not examine the effect of calcium and vitamin D analogs supplementation. Importantly, the validity and robustness of NMA depends not only on the heterogeneity in case of standard pairwise meta-analysis, but also on the inconsistency between direct and indirect contrast estimates. No evidence of inconsistency was found in this NMA. Bisphosphonate plus calcium revealed a significant gain in percent BMD change than calcium alone. The heterogeneity was calculated from the pairwise meta-analysis with four RCTs [31–33, 35]. The sample size, races and bisphosphonates which included ibandronate [31], pamidronate [32], clodronate [33], alendronate [35] were different. These resulted in high heterogeneity which would reduce the quality of evidence. Adding bisphosphonates also showed significant improvement than combination of calcium and vitamin D analogs in both absolute and percent BMD change at the lumbar with high heterogeneity. The heterogeneity was associated with characteristics of samples, different inclusion and exclusion criterion. The quality of evidence downgraded because of high heterogeneity. At this stage, limited information made it difficult to perform further analysis. Thus, we could not ignore the impact of heterogeneity when draw the conclusion. Although bisphosphonate with calcium and vitamin D analogs ranked the best, we did not detect any significant differences between combination use of bisphosphonate and calcium with or without vitamin D analogs from the indirect comparisons. Moreover, indirect comparisons would lead to very low quality of evidence. Only RCT conducted by Fan SL et al. [40, 41] compared bisphosphonate alone with placebo. They found that only two intravenous doses of pamidronate can protect the skeleton from bone loss even 4 years later after transplantation. We could not specify the influence of bisphosphonate monotherapy and access the situation of KTRs with no treatment due to lack of relevant studies.

Included RCTs used BMD as a surrogate marker and did not provide sufficient data to make a polygonal network configuration about the fracture. Also, the association between BMD metrics and fracture risk in KTRs is still controversial. West SL et al. [42] indicated that low BMD was a risk factor for subsequent fracture in patients with pre-dialysis CKD, but data for KTRs are scant. According to KDIGO guideline [7], bone biopsy is reasonable to guide treatment in the first twelve-months after transplantation. However, it is an invasive procedure and most centers lack the expertise to properly process and analyze bone biopsy specimens. Recently, the Fracture Risk Assessment Tool (FRAX) [43] and the spine Trabecular bone score (TBS) [44] were detected as new measurements for KTRs to predict fracture risk. Consequently, surrogate outcomes poorly reflect pathological bone changes. Future trials need to find more specific measurements for detecting mineral and bone disorders in KTRs.

Evidence on other secondary outcomes was limited. There was an unexpected finding from previous reviews [9, 45] that bisphosphonate reduced acute graft rejection moderately. Bisphosphonates could suppress cytokine releasing from activated macrophages to inhibit T-cell function. Its immunomodulatory and anti-inflammatory properties may explain this finding. However, the confidence intervals were wide and ignored the influence of co-interventions. The use of bisphosphonate was limited on account of its nephrotoxicity and development of the adynamic bone disease. We did not find additional bisphosphonate use would increase the occurrence of graft loss and adverse events. Apart from mild gastrointestinal side effects, include RCTs did not report or systematically study serious adverse events. Perazella MA et al. [46] summarized that bisphosphonate nephrotoxicity is infusion time-dependent and dose-dependent. Increasing the time interval between doses can limit its nephrotoxicity. On the current situation, bisphosphonate therapy was well tolerated whereas controversial data on its potency in preventing fracture limited its widespread.

Our analysis updated the previous meta-analysis and conducted a comprehensive search with broad inclusion criteria to maximize available data in this field. Only RCTs that supplied BMD results with g/cm^2^ were included to standardize each comparison, while some studies used different units such as Z-score or T-score. Furthermore, we used GRADE approach to measure the quality of evidence and also performed sensitivity analyses to demonstrate the robustness of estimates. In addition, only adult KTRs were included to offer more reliable evidence and minimize potential bias. To our knowledge, this is the first NMA that took co-intervention (calcium, vitamin D analogs) into account when examining the effect of bisphosphonates and expands on previous meta-analyses as well [9, 45].

However, this NMA still has several limitations including the omission of important methodological details in RCTs and the possibility of reporting biases. Most included studies had a high risk of bias and their impact on results is uncertain. Moreover, some studies only included cadaveric allograft, while some studies excluded patients with diabetes or postmenopausal women. These were risk factors for fracture. Preexisting CKD-MBD, immunosuppression therapy including steroid dosage, CNIs type could also cause bone disease after KT. Because of insufficient information, we could not perform further analysis to identify the influence of relevant factors. These would result in high heterogeneity which may downgrade the quality of evidence as well. Aside from different basic characteristic of the participant, it is unknown if within the drug class of bisphosphonates certain drugs are more favorable than others, and the bisphosphonates regimen (dosage, route, timing, and administration duration) differ among the included studies. We grouped vitamin D, calcitriol, alfacalcidol together as vitamin D analogs and did not distinguish their efficacy. These factors may potentially influence the calculation of BMD between RCTs.

More high-quality RCT is required to determine the optimal therapy for KTRs to prevent fractures with minimal risk for side effects. We also need to find a more correlative measurement than BMD to reflect pathological bone changes in KTRs. Future studies should be powered to show the fracture risk with sufficient follow-up time (≥3 years) and adequate sample sizes, while providing methodological details.

## Conclusion

At this stage, we suggested the additional use of bisphosphonate was well-tolerated and more favorable in KTRs to improve BMD at the lumbar spine and femoral neck. However, evidence to reduce fracture risk is insufficient. Clinicians should take all known safety information and compliance of patients into account when using bisphosphonates. Further studies are needed to support our findings and find optimal treatment option for KTRs.

## Additional file

Additional file 1: Search algorithms. (DOCX 17 kb)

Additional file 2: Risk of bias assessments within studies. (DOCX 675 kb)

Additional file 3: Network plot for secondary outcomes. (DOCX 223 kb)

Additional file 4: Network meta-analysis of secondary outcomes. (DOCX 22 kb)

Additional file 5: Subgroup analysis and sensitivity analysis. (DOCX 17 kb)
