# Supplementary material for: Outcomes of bisphosphonate and its supplements for bone loss in kidney transplant recipients: a systematic review and network meta-analysis
Source: BMC Nephrol. 2018 Oct 19;19:269. doi: 10.1186/s12882-018-1076-1 (PMC6194739; doi:10.1186/s12882-018-1076-1)
Supplement: Supplementary file 1 — Search algorithms. (DOCX 17 kb) [file 12882_2018_1076_MOESM1_ESM.docx]

**Additional file 1. Search algorithms**

#1 MeSH descriptor: [Kidney Transplantation] explode all trees

#2 kidney transplant*

#3 renal transplant*

#4 #1 or #2 or #3

#5 MeSH descriptor: [Disphosphonates] explode all trees

#6 alendron*

#7 clodron*

#8 etidron*

#9 ibandron*

#10 Incadron*

#11 Medron*

#12 Olpadron*

#13 Pamidron*

#14 Risedron*

#15 Tiludron*

#16 Zoledron*

#17 bisphosphonat*

#18 disphosphonat*

#19 #5 or #6 or #7 or #8 or #9 or #10 or #11 or #12 or #13 or #14 or #15 or #16 or #17 or#18

#20 #4 and #19
