# Supplementary material for: Outcomes of bisphosphonate and its supplements for bone loss in kidney transplant recipients: a systematic review and network meta-analysis
Source: BMC Nephrol. 2018 Oct 19;19:269. doi: 10.1186/s12882-018-1076-1 (PMC6194739; doi:10.1186/s12882-018-1076-1)
Supplement: Supplementary file 2 — Risk of bias assessments within studies. (DOCX 675 kb) [file 12882_2018_1076_MOESM2_ESM.docx]

**Additional file 2. Risk of bias assessments within studies**

a. Risk of bias graph: review authors' judgements about each risk of bias item presented as percentages across all included studies.
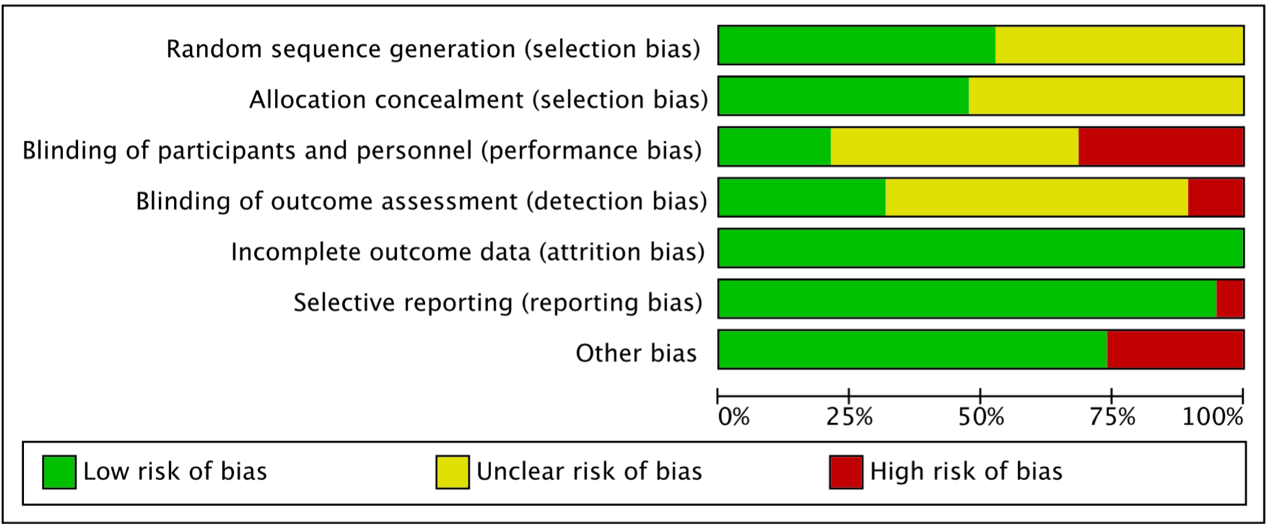


b. Study-level risk of bias.
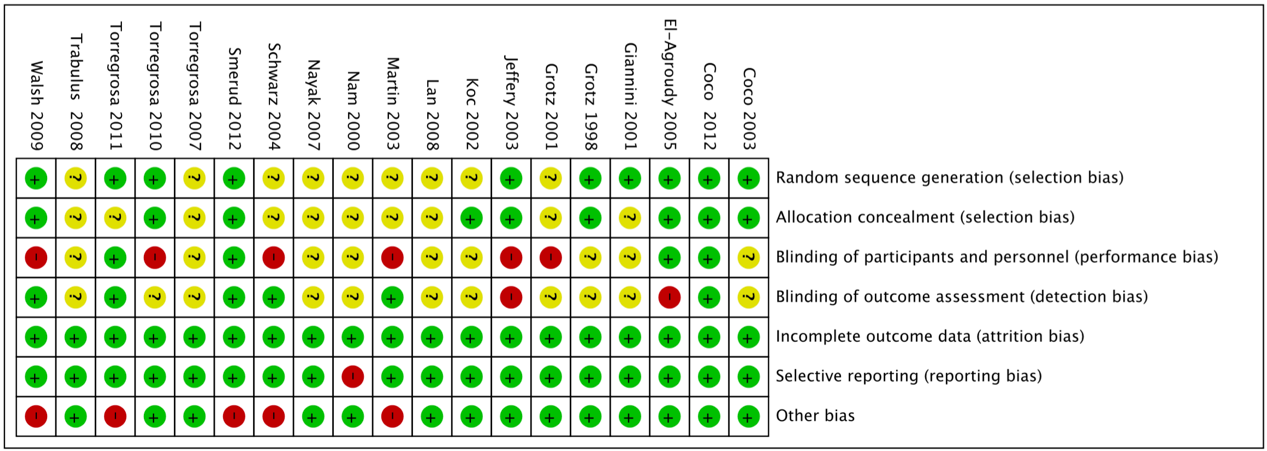


c. Publication bias assessed via funnel plots for absolute BMD change at the femoral neck between individual treatment.


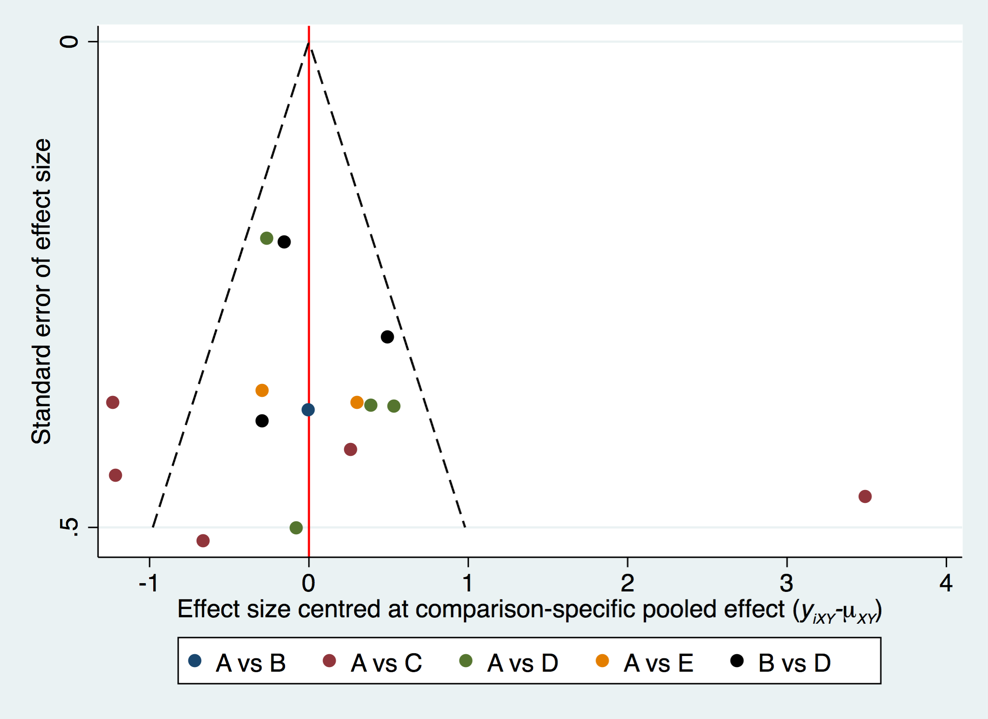


A=Bisphosphonate + calcium; B=Bisphosphonate + calcium + vitamin D analogs; C=Calcium; D=Calcium + vitamin D analogs; E=Calcitonin + calcium.

d. Publication bias assessed via funnel plots for absolute BMD change at the lumbar spine between individual treatment.


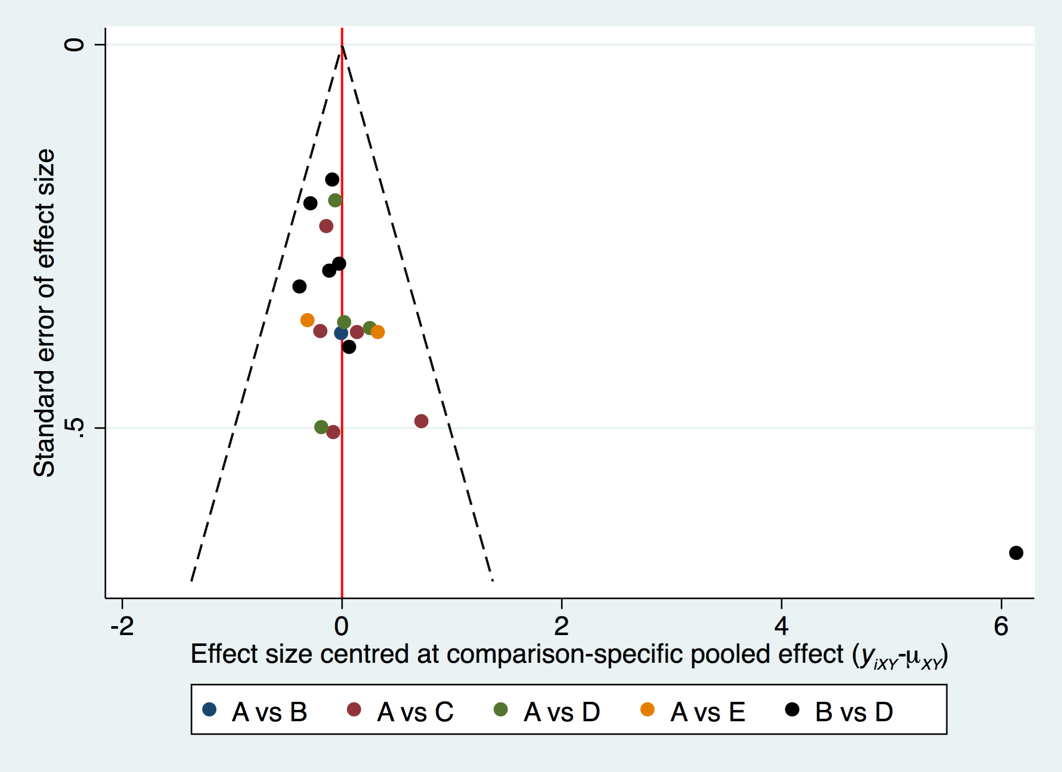


A=Bisphosphonate + calcium; B=Bisphosphonate + calcium + vitamin D analogs; C=Calcium; D=Calcium + vitamin D analogs; E=Calcitonin + calcium.

e. Publication bias assessed via funnel plots for adverse events between individual treatment.


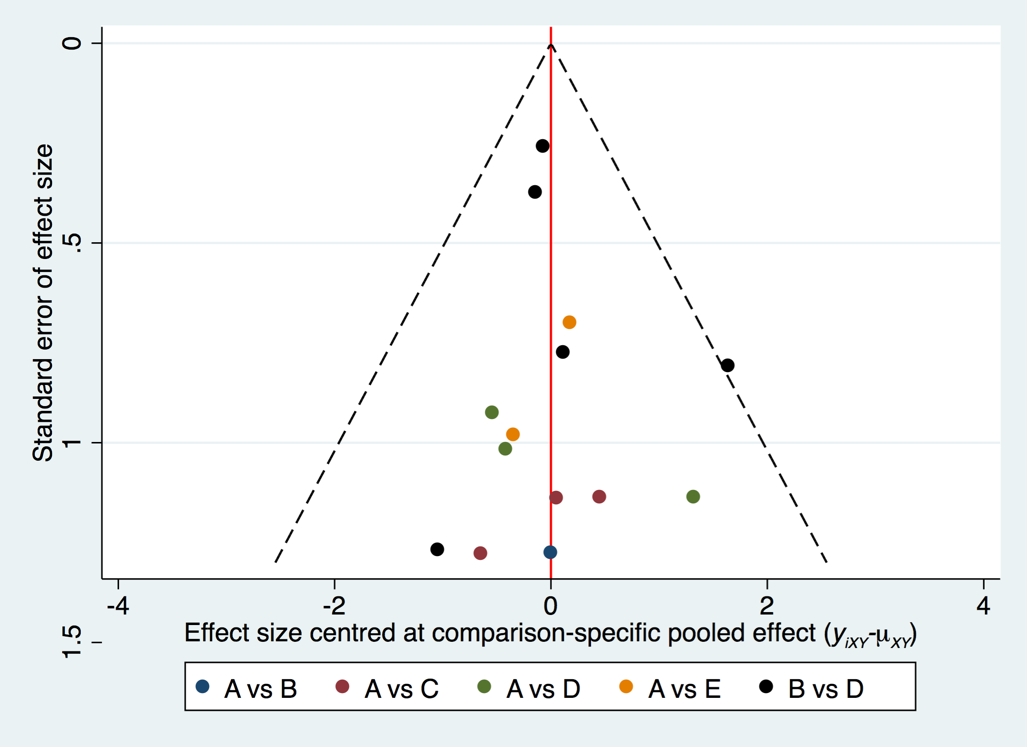


A=Bisphosphonate + calcium; B=Bisphosphonate + calcium + vitamin D analogs; C=Calcium; D=Calcium + vitamin D analogs; E=Calcitonin + calcium.

Small-study effects assessed via comparison-adjusted network funnel plots. In this presentation, all studies are centered on the summary effect estimate of their respective comparisons [μXY (logOR for present study)] which is represented by the vertical red line. Individual study-level effect size is represented by yiXY [where X and Y are two study agents]. The green line represents linear regression of the comparison specific differences yi - μXY on the standard error of yi. Outer dotted lines indicate the triangular region within which 95% of studies are expected to lie in the absence of both biases and heterogeneity (logOR ± 1.96*standard error). Please note that this is drawn only for comparisons with 2 or more studies.
