# Supplementary material for: Outcomes of bisphosphonate and its supplements for bone loss in kidney transplant recipients: a systematic review and network meta-analysis
Source: BMC Nephrol. 2018 Oct 19;19:269. doi: 10.1186/s12882-018-1076-1 (PMC6194739; doi:10.1186/s12882-018-1076-1)
Supplement: Supplementary file 3 — Network plot for secondary outcomes. (DOCX 223 kb) [file 12882_2018_1076_MOESM3_ESM.docx]

**Additional file 3. Network plot for secondary outcomes**


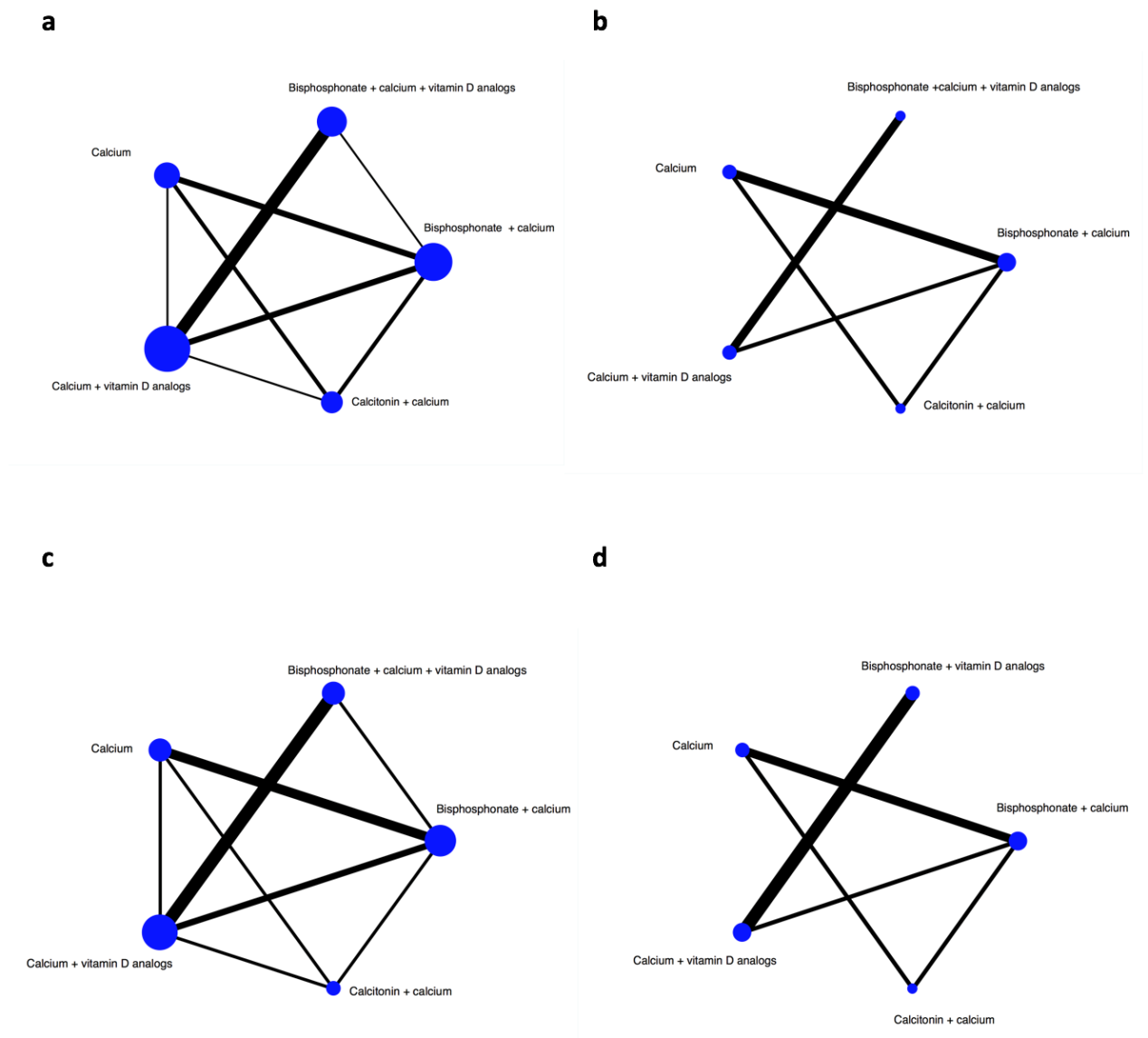


Network of eligible comparisons for secondary outcome

The width of the lines is proportional to the number of trials comparing every pair of treatments, and the size of every circle is proportional to the number of randomly assigned participants (sample size). (a) adverse events; (b) all-cause mortality; (c) biopsy-proven acute rejections; (d) graft loss.
