# Supplementary material for: Outcomes of bisphosphonate and its supplements for bone loss in kidney transplant recipients: a systematic review and network meta-analysis
Source: BMC Nephrol. 2018 Oct 19;19:269. doi: 10.1186/s12882-018-1076-1 (PMC6194739; doi:10.1186/s12882-018-1076-1)
Supplement: Supplementary file 4 — Network meta-analysis of secondary outcomes. (DOCX 22 kb) [file 12882_2018_1076_MOESM4_ESM.docx]

**Additional file 4. Network meta-analysis of secondary outcomes**

| Comparisons | No. of events (participants) | Pairwise meta-analysis odds ratios (95% CI) | Network meta-analysis odds ratios (95% CrI) | Heterogeneity I^2^ | CoChran's Q p-Value | Quality of evidence |
| --- | --- | --- | --- | --- | --- | --- |
| Adverse events (760) | | | | | | |
| Bis+Ca vs. Bis+Ca+Vit D | 1 (30) | - | 0.28 (0.04, 1.75) | - | - | Low |
| Bis+Ca vs. Ca | 3 (140) | **5.410 (1.148, 25.492)** | 0.17 (0.02, 1.13) | 0.00% | 0.653 | Low |
| Bis+Ca vs. Ca+Vit D | 3 (185) | 1.871 (0.582, 6.010) | 0.43 (0.08, 2.17) | 19.00% | 0.291 | Medium |
| Ca+Calcitonin vs. Bis+Ca | - | - | 0.78 (0.12, 4.80) | - | - | Very low |
| Bis+Ca+Vit D vs. Ca | - | - | 0.60 (0.04, 8.19) | - | - | Very low |
| Ca+Vit D vs. Bis+Ca+Vit D | 6 (444) | 1.129 (0.778, 1.637) | 0.66 (0.21, 1.90) | 15.80% | 0.312 | Medium |
| Ca+Calcitonin vs. Bis+Ca+Vit D | - | - | 0.22 (0.02, 2.35) | - | - | Very low |
| Ca+Vit D vs. Ca | 1 (30) | - | 0.40 (0.03, 4.41) | - | - | Low |
| Ca+Calcitonin vs. Ca | 2 (61) | 0.209 (0.034, 1.284) | 0.13 (0.01, 1.08) | 0.00% | 0.547 | Medium |
| Ca+Calcitonin vs. Ca+Vit D | 1 (30) | - | 0.33 (0.03, 3.05) | - | - | Low |
| All-cause mortality (473) | | | | | | |
| Bis+Ca vs. Bis+Ca+Vit D | - | - | 0.02 (0.00, 2.70) |  |  | Very low |
| Ca vs. Bis+Ca | 2 (110) | 1.799 (0.367, 8.849) | 0.46 (0.04, 4.23) | 0.00% | 0.717 | Medium |
| Bis+Ca vs. Ca+Vit D | 1 (117) | - | 0.17 (0.00, 10.70) | - | - | Low |
| Bis+Ca vs. Ca+Calcitonin | 1 (31) | - | 0.52 (0.00, 38.11) | - | - | Low |
| Ca vs. Bis+Ca+Vit D | - | - | 0.01 (0.00, 1.92) | - | - | Very low |
| Ca+Vit D vs. Bis+Ca+Vit D | 2 (230) | 5.263 (0.604, 45.456) | 0.13 (0.00, 1.65) | 0.00% | 0.707 | Medium |
| Ca+Calcitonin vs. Bis+Ca+Vit D | - | - | 0.04 (0.00, 231.11) | - | - | Very low |
| Ca vs. Ca+Vit D | - | - | 0.07 (0.00, 8.69) | - | - | Very low |
| Ca vs. Ca+Calcitonin | 1 (31) | - | 0.24 (0.00, 12.15) | - | - | Low |
| Ca+Calcitonin vs. Ca+Vit D | - | - | 0.35 (0.00, 1575.32) | - | - | Very low |
| Acute rejection (463) | | | | | | |
| Bis+Ca vs. Bis+Ca+Vit D | - | - | 0.67 (0.16, 2.99) | - | - | Very low |
| Ca vs. Bis+Ca | 3 (122) | 1.715 (0.840, 3.509) | 0.51 (0.15, 2.26) | 0.00% | 0.139 | Medium |
| Bis+Ca vs. Ca+Vit D | 2 (63) | 1.514 (0.522, 4.392) | 0.92 (0.22, 3.92) | 19.50% | 0.265 | Medium |
| Ca+Calcitonin vs. Bis+Ca | 1 (30) | - | 0.96 (0.13, 9.34) | - | - | Low |
| Ca vs. Bis+Ca+Vit D | - | - | 0.35 (0.08, 1.84) | - | - | Very low |
| Ca+Vit D vs. Bis+Ca+Vit D | 4 (299) | 0.827 (0.529, 1.293) | 0.72 (0.30, 1.97) | 0.00% | 0.696 | Medium |
| Ca+Calcitonin vs. Bis+Ca+Vit D | - | - | 0.65 (0.08, 7.24) | - | - | Very low |
| Ca vs. Ca+Vit D | 1 (30) | - | 0.49 (0.10, 2.36) | - | - | Low |
| Ca vs. Ca+Calcitonin | 1 (30) | - | 0.52 (0.06, 4.59) | - | - | Low |
| Ca+Calcitonin vs. Ca+Vit D | 1 (30) | - | 0.91 (0.11, 8.84) | - | - | Low |
| Graft loss (478) | | | | | | |
| Bis+Ca vs. Bis+Ca+Vit D | - | - | 0.18 (0.00, 11.02) | - | - | Very low |
| Ca vs. Bis+Ca | 2 (109) | 0.993 (0.192, 5.155) | 0.91 (0.08, 11.24) | 53.80% | 0.141 | Medium |
| Bis+Ca vs. Ca+Vit D | 1 (117) | - | 0.18 (0.00, 8.16) | - | - | Low |
| Bis+Ca vs. Ca+Calcitonin | - | - | 0.15 (0.00, 6.56) | - | - | Very low |
| Ca vs. Bis+Ca+Vit D | - | - | 0.16 (0.00, 19.33) | - | - | Very low |
| Bis+Ca+Vit D vs. Ca+Vit D | 3 (236) | 0.969 (0.473, 1.983) | 1.01 (0.22, 4.60) | 0.00% | 0.923 | Medium |
| Bis+Ca+Vit D vs. Ca+Calcitonin | - | - | 0.77 (0.00, 1899.07) | - | - | Very low |
| Ca vs. Ca+Vit D | - | - | 0.16 (0.00, 15.21) | - | - | Very low |
| Ca vs. Ca+Calcitonin | 1 (30) | - | 0.14 (0.00, 6.37) | - | - | Low |
| Ca+Vit D vs. Ca+Calcitonin | - | - | 0.78 (0.00, 1530.02) | - | - | Very low |

Bis: bisphosphonate; Ca: calcium; Vit D: Vitmin D analogs; 95% CI: 95% Confidence Intervals; 95% CrI: 95% Credible Intervals. The odds ratios with 95% CI or 95% CrI was used for categorical outcomes. Significant results are in bold. The Grading of Recommendations Assessment, Development and Evaluation (GRADE) approach specific to NMA served to assess the certainty in the evidence (quality of evidence) associated with specific comparisons, including direct, indirect, and final network meta-analysis estimates. The confidence assessment addressed the risk of bias (in individual studies), imprecision, inconsistency (heterogeneity in estimates of effect across studies), indirectness, and publication bias.
