# Supplementary material for: Outcomes of bisphosphonate and its supplements for bone loss in kidney transplant recipients: a systematic review and network meta-analysis
Source: BMC Nephrol. 2018 Oct 19;19:269. doi: 10.1186/s12882-018-1076-1 (PMC6194739; doi:10.1186/s12882-018-1076-1)
Supplement: Supplementary file 5 — Subgroup analysis and sensitivity analysis. (DOCX 17 kb) [file 12882_2018_1076_MOESM5_ESM.docx]

**Additional file 5. Subgroup analysis and sensitivity analysis**

| Comparisons | No. of events (participants) | Network meta-analysis mean differences (95% CrI) |
| --- | --- | --- |
| absolute BMD change at the lumbar spine for 12 months (607) | | |
| Bis+Ca vs. Bis+Ca+Vit D | 1 (29) | -0.02 (-0.08, 0.04) |
| Bis+Ca vs. Ca | 4 (147) | **0.06 (0.01, 0.10)** |
| Bis+Ca vs. Ca+Vit D | 4 (176) | 0.02 (-0.03, 0.06) |
| Bis+Ca vs. Ca+Calcitonin | 2 (61) | 0.08 (-0.05, 0.21) |
| Bis+Ca+Vit D vs. Ca | - | **0.07 (0.01, 0.15)** |
| Bis+Ca+Vit D vs. Ca+Vit D | 5 (297) | **0.03 (0.00, 0.07)** |
| Bis+Ca+Vit D vs. Ca+Calcitonin | - | 0.09 (-0.05, 0.24) |
| Ca vs. Ca+Vit D | 2 (46) | -0.04 (-0.11, 0.03) |
| Ca vs. Ca+Calcitonin | 2 (60) | 0.02 (-0.11, 0.15) |
| Ca+Vit D vs. Ca+Calcitonin | 1 (30) | 0.06 (-0.09, 0.20) |
| absolute BMD change at the femoral neck for 12 months (377) | | |
| Bis+Ca vs. Bis+Ca+Vit D | 1 (29) | 0.00 (-0.31, 0.33) |
| Bis+Ca vs. Ca | 4 (147) | **0.23 (0.02, 0.46)** |
| Bis+Ca vs. Ca+Vit D | 4 (176) | 0.07 (-0.17, 0.30) |
| Bis+Ca vs. Ca+Calcitonin | 2 (61) | 0.09 (-0.21, 0.39) |
| Bis+Ca+Vit D vs. Ca | - | 0.23 (-0.14, 0.55) |
| Bis+Ca+Vit D vs. Ca+Vit D | 2 (67) | 0.06 (-0.18, 0.30) |
| Bis+Ca+Vit D vs. Ca+Calcitonin | - | 0.09 (-0.32, 0.50) |
| Ca vs. Ca+Vit D | 2 (46) | -0.16 (-0.41, 0.10) |
| Ca vs. Ca+Calcitonin | 2 (60) | -0.14 (-0.46, 0.18) |
| Ca+Vit D vs. Ca+Calcitonin | 1 (30) | 0.03 (-0.34, 0.39) |

Bis: bisphosphonate; Ca: calcium; Vit D: Vitmin D analogs; 95% CrI = 95% Credible Intervals. The mean difference with 95% CrI was used for continuous outcomes. Significant results are in bold.
